# A prospective evaluation on external jugular vein cut-down approach for TIVAD implantation

**DOI:** 10.1186/s12957-015-0663-x

**Published:** 2015-08-12

**Authors:** Giuseppe Cavallaro, Olga Iorio, Angelo Iossa, Francesco De Angelis, Marcello Avallone, Matteo Massaro, Consalvo Mattia, Gianfranco Silecchia

**Affiliations:** Department of Medico-Surgical Sciences and Biotechnologies, Sapienza University, Rome, Italy

## Abstract

**Background:**

Totally implantable venous access devices can be implanted both by percutaneous approaches and by surgical approaches with cephalic vein or external jugular vein cut-down techniques that are related to low intraoperative complication rates. The authors report a prospective evaluation of 83 consecutive external jugular vein cut-down approaches for totally implantable venous access devices implantation.

**Methods:**

Eighty three consecutive patients (28 M, 55 F, mean age 54.2) suffering from solid tumors (58) or hematologic diseases (25) were consecutively submitted to totally implantable venous access devices insertion through external jugular vein cut-down approach (75 on right side, 8 on left side).

**Results:**

All devices were surgically implanted; no instances of intraoperative complications were detected. After a minimum follow-up of 150 days, only one case of wound hematoma and one case of device malfunction due to incorrect catheter angulation were noted.

Postoperative patient satisfaction was evaluated by the use of specific questionnaire that demonstrated a good satisfaction and compliance (92.8 %) of patients with implanted devices.

**Conclusions:**

Despite the lack of controlled studies comparing external jugular vein cut-down approach vs other approaches, this approach should be considered as a tool for long-term central vein catheters positioning, both as an alternative and for primary approach.

## Background

Totally implantable venous access devices (TIVADs) are usually inserted and utilized in case of long-term therapies and nutritional support in oncologic patients [1, 2]. These devices can typically be implanted through different ways. The percutaneous approach is the most used technique and can be performed under ultrasound guidance or “blind” puncture, via the subclavian or internal jugular vein (using the Seldinger technique) [1]; nevertheless, this approach is still affected by several and potentially severe complications, like pneumothorax, hemothorax, vascular, or brachial plexus injuries, occurring in up to 12 % of patients [3, 4]. On the other hand, cephalic vein cut-down (CVCD) technique appears to be effective and not affected by these severe complications [3–5] but is reported to fail in 6–30 % of cases, mainly due to anatomic variations in cephalic vein course or caliber. In such cases, several authors report high success rates in positioning TIVAD via the external jugular vein (EJV) by cut-down approach [3–5].

The authors report a prospective evaluation of 83 consecutive EJV cut-down (EJV-CD) approach procedures for TIVAD implantation, evaluating success rates, intraoperative and postoperative complications, and patient satisfaction.

## Methods

From January 2014 to June 2014, 83 consecutive patients (28 M, 55 F, mean age 54.2) suffering from solid tumors (58) or hematologic diseases (25) were consecutively submitted to TIVAD implantation through EJV-CD approach (75 on right side, 8 on left side). Patient characteristics, oncological diagnosis, and comorbidities are summarized on Table 1. All devices used were Celsite® ST201F (B.Braun Medical, 92107 Boulogne Cedex – France), with 6.5 Fr. silicone catheter, and 0.035-in. guidewire.

**Table 1:** Patients demographics and characteristics

Gender (M/F)	28/55
Mean age	54.8 ± 16.4
Comorbidities	
Diabetes	18
Morbid obesity (BMI >35)	4
COPD	7
Cardiovascular disease	21
Hypertension	42
Oncologic diagnosis	
Gastrointestinal	31
Breast	12
Lung	9
Hematological	25
Other	6

All patients received single-shot preoperative antibiotic prophylaxy 30 min before surgery.

### Implantation technique

**Fig. 1:**
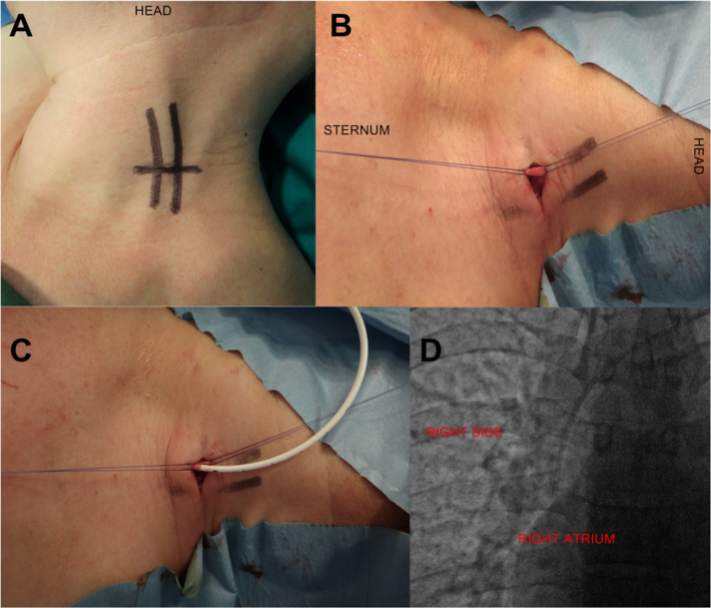
Technique of TIVAD implantation, on the left side. The head is rotated to the right. **a** The route of the left EJV. **b** The EJV is identified. **c** The catheter is inserted into the vein. **d** Intraoperative fluoroscopy reveals the good positioning of the catheter tip just above the right atrium

All procedures were performed by the same surgeon (GC) having large experience in TIVAD implantation. All devices were implanted in the operating theater under fluoroscopic control, and patients were treated and monitored in a day hospital setting. Seventy five TIVADs were inserted on the right side, and, due to right breast or lung cancer or to previous TIVAD or other central venous catheter implantation on the right side, the other eight were implanted on the left side. The route of the external jugular vein was identified and marked by a permanent marker prior to surgery, by placing the patient in mild Trendelemburg position and using the Valsalva maneuver (Fig. 1a). After local anesthesia (2 % solution of mepivacaine hydrocloride), 1.5-cm skin incision was performed on the neck skin. After careful dissection of subcutaneous space, the EJV was identified and exposed (Fig. 1b) Then, the catheter was inserted (Fig. 1c) into the vein for about 15 cm. The correct positioning of the catheter tip was controlled by fluoroscopy (Fig. 1d). So the catheter was connected to the port (placed in a subfascial pocket on the pectoralis muscle) through a subcutaneous tunnel (using the specific device present in the TIVAD sterile package). In 14 cases (16.9 %), due to difficult progression of the catheter through the vein lumen, a J-tip guidewire inserted into the catheter lumen was used to facilitate the progression. At the end of the procedure, a test puncture was performed to check patency and flow through the system with sodium heparin solution (5000 IU in 10 ml of 0.9 % saline solution).

## Results

Intraoperative and postoperative results and complications are summarized in Table 2. All TIVADs were surgically implanted, with no need of percutaneous access to sublcavian or internal jugular veins. We found no differences in operative time between left and right side. No instances of intraoperative complications, such as pneumothorax, hemotorax, injuries to great vessels, or brachial plexus were detected. After a minimum follow-up of 150 days only one case of wound hematoma and one case of device malfunction due to excessive catheter angulation requiring surgical revision of the catheter were noted. No cases of deep venous thrombosis or catheter rupture were noted.

**Table 2:** Intraoperative and postoperative results

Side of implantation (R/L)	75/8
Operative time	
Right side	18.2 min (range 11–36)
Left side	19.4 min (range 14–35)
Success rate	83/83 (100 %)
Use of guidewire	14/83 (16.9 %)
Intraoperative complications	0/83 (0 %)
Postoperatve complications	2/80 (2.5 %)
Wound hematoma	1
Device malfunction	1

Postoperative patients satisfaction was evaluated by the use of specific questionnaire on postoperative satisfaction after TIVAD implantation (Table 3) [6]. The reported results demonstrated a good satisfaction about the surgical procedure and outcome and a good compliance (92.8 %) of patients with implanted devices.

**Table 3:** Patient questionnaire to assess changes in TIVAD implantation (taken from: Faigo JL. Quality of life and Patients Satisfaction. In: Di Carlo I, Biffi R. Totally implantable venous access devices: management in mid and long-term clinical settings. Springer Eds, 2012; 37: 265–268. DOI: 10.1007/978-88-470-2373-4. ISBN: 978-88-470-2372-7)

Table query	Yes (%)	No (%)
Were you well informed about the surgical procedure?	79 (95.1 %)	4 (4.9 %)
Did you feel supported during the operation?	76 (91.5 %)	9 (8.5 %)
Was the procedure painful?	11 (13.2 %)	72 (86.8 %)
Did the procedure take too long?	10 (12.0 %)	73 (88.0 %)
Were you provided with adequate information about the catheter?	80 (96.4 %)	3 (3.6 %)
Would you repeat the procedure under local anesthesia?	75 (90.4 %)	8 (9.6 %)
Would you repeat the procedure in a day hospital setting?	81 (97.5 %)	2 (2.5 %)
Would you counsel a friend or relative to undergo such a procedure under the same conditions?	76 (91.5 %)	9 (8.5 %)
Were your preoperative fears and expectations realized?	15 (18.1 %)	68 (81.9 %)
Did the TIVAD affect your daily living?	6 (7.2 %)	77 (92.8 %)
Did the TIVAD facilitate the treatment?	81 (97.5 %)	2 (2.5 %)

## Discussion

EJV approach represents an effective way for accessing the central venous system, reducing severe complications related to deep vein puncture: pneumothorax, hemothorax, arterial or nerve injuries, and deep venous thrombosis [7–12], and it is currently used for many kinds of central venous catheters (mainly dyalisis and Groshong catheters) especially in pediatric patients [13–17], reporting low complication rates and higher success rates, even in emergency and intensive care settings [18–22].

Nevertheless, only few reports dealing EJV approach for TIVAD implantation exist.

However, a recent literature review [23] and other previous studies [3, 24] support the evidence that approaches to the EJV (both cut-down and percutaneous) are safe and effective even for TIVAD implantation.

The present prospective study on 83 primary EJV-CD approaches for TIVAD implantation report a 100 % success rate and a very low incidence of complications (2.4 %); furthermore, only one of these complications appears related to the specific technique itself and specifically to an incorrect angulation of the catheter when entering the EJV. This low incidence of complications is due to a totally surgical approach, with no direct vein puncture, and to the easy dissection and identification of the vein that runs superficially and can be easily detected preoperatively, by placing the patient in mild Trendelemburg position and after Valsalva maneuver.

In 14 patients, due to a difficult progression of the catheter from the EJV to the right atrium, we used a J-tip guidewire to ensure the correct catheter positioning. Difficulties in catheter progression through the vein route are mainly due to a sharp angulation between the EJV and the subclavian vein, occurring in about 12 % of patients, as recently demonstrated by radiological evaluations [25, 26]. However, the simple use of the guidewire within the catheter lumen allowed a correct progression of the catheter until the right atrium.

Despite the high success and low complication rates reported by this prospective evaluation, as well as by previous studies, there are still several controversies about the potential role of EJV-CD as primary choice for TIVAD implantation. First, the need to perform two separate incisions (one for EJV catheterization and one for the subfascial pocket for the Port) and second, the need to make a subcutaneous tunnel for the catheter and the consequent risk to create an obstacle (angulation) to the catheter flow, just near the EJV inlet. This occurrence can be avoided by creating a “gentle” shape to the catheter curve near the EJV inlet, and actually, there is no evidence that the subcutaneous tunnel itself may be related to any complication or TIVAD malfunction. Another criticism related to the presence of the tunneled catheter is the possible discomfort of patients about the presence of the catheter in subcutaneous tissues of the neck and above the clavicule that can give a “foreign body” sensation and perceived as not esthetic. For these reasons, we proposed, 30 days after surgery, a specific patient satisfaction questionnaire designed for TIVAD implantation [6]. Results of this questionnaire, summarized in Table 3, reflect a good patient satisfaction and compliance (in 92.8 % of cases) with TIVAD implanted through the EJV, thus encouraging us to continue in this experience.

## Conclusions

In conclusion, this prospective evaluation reports a 100 % success rate in TIVAD implantation through EJV-CD approach, with very low incidence of complications and high levels of patient satisfaction, demonstrated by a dedicated questionnaire. So, despite the lack of controlled studies comparing EJV-CD approach vs other approaches that cannot lead to definitive conclusions about the “gold standard” technique, we can say that this approach should be considered at least as a tool for long-term central vein catheters positioning, both as an alternative and for primary approach.
